# Multiple QTL for Horticultural Traits and Quantitative Resistance to *Phytophthora infestans* Linked on *Solanum habrochaites* Chromosome 11

**DOI:** 10.1534/g3.114.014654

**Published:** 2014-12-12

**Authors:** J. Erron Haggard, Emily B. Johnson, Dina A. St. Clair

**Affiliations:** Plant Sciences Department, University of California-Davis, Davis, California 95616

**Keywords:** tomato, *Solanum lycopersicum*, introgression, QTL mapping, linkage drag, late blight disease

## Abstract

Previously, a *Phytophthora infestans* resistance QTL from *Solanum habrochaites* chromosome 11 was introgressed into cultivated tomato (*S. lycopersicum*). Fine mapping of this resistance QTL using near-isogenic lines (NILs) revealed some co-located QTL with undesirable effects on plant size, canopy density, and fruit size traits. Subsequently, higher-resolution mapping with sub-NILs detected multiple *P. infestans* resistance QTL within this 9.4-cM region of chromosome 11. In our present study, these same sub-NILs were also evaluated for 17 horticultural traits, including yield, maturity, fruit size and shape, fruit quality, and plant architecture traits in replicated field experiments over 2 years. The horticultural trait QTL originally detected by fine mapping each fractionated into two or more QTL at higher resolution. A total of 34 QTL were detected across all traits, with 14% exhibiting significant QTL × environment interactions (QTL × E). QTL for many traits were co-located, suggesting either pleiotropic effects or tight linkage among genes controlling these traits. Recombination in the pericentromeric region of the introgression between markers TG147 and At4g10050 was suppressed to approximately 29.7 Mbp per cM, relative to the genomewide average of 750 kbp per cM. The genetic architecture of many of the horticultural and *P. infestans* resistance traits that mapped within this chromosome 11 *S. habrochaites* region is complex. Complicating factors included fractionation of QTL, pleiotropy or tight linkage of QTL for multiple traits, pericentromeric chromosomal location(s), and/or QTL × E. High-resolution mapping of QTL in this region would be needed to determine which specific target QTL could be useful in breeding cultivated tomato.

Introgressions from wild species are important resources for broadening the genetic base of cultivated species, particularly for traits where little variability currently exists. This is certainly the case for cultivated tomato (*Solanum lycopersicum*), an economically important vegetable crop species with limited genetic variability (Park *et al.* 2004). The genetic diversity of tomato has been augmented through introgression of alleles from several closely related wild species (Labate and Robertson 2012). One of these species, *Solanum habrochaites*, has been an important source of favorable alleles for horticultural traits such as yield, fruit size, and fruit quality (Ben Chaim *et al.* 2006; Bernacchi *et al.* 1998b; Mathieu *et al.* 2009; Monforte and Tanksley 2000; Yates *et al.* 2004). This wild species also contains genes for resistance to major tomato diseases such as late blight, bacterial canker, gray mold, and early blight (Brouwer *et al.* 2004; Brouwer and St. Clair 2004; Coaker and Francis 2004; Finkers *et al.* 2007; Foolad *et al.* 2002; Zhang *et al.* 2003).

In cultivated tomato, genetic diversity is particularly lacking for resistance to late blight disease caused by *Phytophthora infestans* (Foolad *et al.* 2008). Late blight is an economically important and devastating disease of both tomato and potato because it results in approximately $5 billion in annual crop losses and chemical control costs (Judelson and Blanco 2005; Nowicki *et al.* 2012).

*S. habrochaites* has genetic resistance to *P. infestans*. QTL for quantitative resistance to *P. infestans* from *S. habrochaites* have been mapped on each of tomato’s 12 chromosomes (Brouwer *et al.* 2004). Three of these QTL (on chromosomes 4, 5, and 11) were then fine-mapped by Brouwer and St.Clair (2004) using near-isogenic lines (NILs). QTL affecting horticultural traits including plant height, plant shape, maturity, yield, and fruit size were co-located and/or linked with each of these resistance QTL, suggesting the potential for linkage drag in crosses between *S. lycopersicum* and *S. habrochaites*.

Subsequently, we mapped the QTL on *S. habrochaites* chromosome 11 at higher resolution using sub-NILs and detected multiple closely linked QTL controlling both foliar and stem resistance to *P. infestans* within a 9.4-cM region (Johnson *et al.* 2012). To gain a better understanding of the genetic basis of QTL controlling horticultural traits and their linkage relationships with QTL for resistance to *P. infestans*, we used this same set of sub-NILs in the present study to map loci controlling horticultural traits and determine linkage relationships among them and with *P. infestans* resistance QTL. We also sought to identify useful breeding material with improved late blight resistance in this set of sub-NILs.

In the present study, we further investigated the *P. infestans* resistance QTL *lb11* region identified by Brouwer and St. Clair (2004), conferred by a *S. habrochaites* introgression on tomato chromosome 11 as a potential source of useful quantitative resistance to late blight disease of tomato. Specifically, our goals in this study were to: assess the effects and extent of linkage drag of QTL controlling horticultural traits with *P. infestans* resistance QTL on *S. habrochaites* chromosome 11; identify markers closely linked to *P. infestans* resistance QTL and to positive alleles at horticultural QTL to facilitate MAS breeding; and identify potentially useful breeding lines for future breeding of tomato cultivars with improved quantitative resistance to late blight disease.

## Materials and Methods

### Plant materials, genotyping, and marker-assisted selection

We developed a set of sub-near-isogenic lines (sub-NILs) in *S. lycopersicum* for a chromosome 11 introgression containing resistance QTL from *P. infestans*-resistant *S. habrochaites* accession LA2099 via marker-assisted selection during backcrossing and selfing generations, as described by Johnson *et al.* (2012). Methods used for genomic DNA extractions, genotyping with chromosome 11 PCR-based markers (SCAR, CAPS, and SSR), primer sequences, enzymatic reaction conditions, and restriction enzymes used for each marker were described by Johnson *et al.* (2012).

We genotyped 1902 BC_6_S_1_ progeny to identify recombinant sub-NIL progeny for the chromosome 11 introgression from *S. habrochaites*; of these progeny, a subset of 852 progeny (150 recombinant, 702 nonrecombinant) was used to construct a linkage map for the introgressed region (see *Linkage and QTL mapping* below). Heterozygous recombinant BC_6_S_1_ individuals were allowed to self-pollinate and progeny were marker-selected to obtain homozygous BC_6_S_2_ sub-NILs. These plants underwent self-pollination to obtain ample BC_6_S_3_ seed for replicated field experiments. We evaluated 62 BC_6_S_3_ sub-NILs in the 2009 field experiments. In the 2010 field experiments, a subset of 42 of the 62 sub-NILs was evaluated to allow increased replication per location while reducing genetic redundancy, as explained previously by Johnson *et al.* (2012). Graphical marker genotypes for the 62 selected BC_6_S_3_ sub-NILs used in field experiments for the present study are presented in Supporting Information, Table S1.

### Field experimental design and procedures

The chromosome 11 BC_6_S_3_ sub-NILs and the parental NIL from which they were derived (subsequently referred to as NIL11) were evaluated in replicated experiments at field locations in Salinas, California (designated as locations 1 and 2) and in Davis, California (locations 3 and 4) over 2 yr. Summer and early fall in Salinas are generally cool and humid, which is conducive to late blight disease development, whereas Davis summers are warm and dry, with no rain, as is typical of California’s Central Valley tomato production areas. Additional information about field sites has been described by Haggard *et al.* (2013).

Seedlings were grown in a greenhouse for 6 weeks and then transplanted into the field locations. Sixty-five genotypes (NIL11, 62 sub-NILs for chromosome 11, and two processing tomato cultivars, E6203 and Hypeel 45) and 45 genotypes (NIL11, 42 sub-NILs, Hypeel 45, and E6203) were included in the 2009 and 2010 experiments, respectively. Experiments were arranged in randomized complete block design (RCBD). For both years, one plot per genotype per block was included, except for controls, for which there were two plots per block. In 2009, three blocks per location were used. In 2010, use of a reduced number of 42 sub-NILs enabled replication to be increased to five blocks in locations 1 and 2 and to four blocks in locations 3 and 4. At each of the four locations, each plot consisted of five plants spaced 0.30 m apart in rows separated by 1.02 m in locations 1 and 2, and by 1.52 m in locations 3 and 4. Border rows and plots with the cultivar E6203 surrounded each experiment at each location to reduce edge effects on the experimental plots. Standard horticultural field practices for processing tomato were used at all locations. Locations 1 and 2 were sprinkler-irrigated, whereas locations 3 and 4 were furrow-irrigated, as needed.

### Phenotypic trait evaluations

All traits were evaluated on a per-plot basis, as described by Haggard *et al.* (2013). Vegetative horticultural traits were evaluated in all four locations. Late blight disease was only evaluated in Salinas (locations 1 and 2) because, as expected, this disease did not occur in Davis due to typical warm, dry summer conditions. Reproductive traits were only evaluated at Davis (locations 3 and 4) due to logistics of timely sampling of ripe fruit. Vegetative horticultural traits measured were plant height (H) and width (W) in cm, canopy density (CD; visual rating scale, 1 = very sparse to 5 = very dense), and plant habit (HAB; visual rating scale, 1 = prostrate to 5 = very upright). H, W, CD, and HAB were obtained at both locations at 71 and 46 days after planting (DAP) in 2009 and 2010, respectively. At locations 3 and 4, these traits were evaluated at 80 DAP in 2009 and at 68 (H and W) and 73 (CD and HAB) DAP in 2010. From plant height and width, two secondary traits were derived, plant size (SZ; product of height × width) and plant shape (SH; ratio of height to width). The reproductive horticultural traits measured or scored were as follows. DAP to maturity was evaluated at two stages of maturity: when each plant in the plot had its first ripe fruit (DAP1st) and when 50% of fruit in a plot were ripe (DAP50). Weight of 30 ripe fruit was evaluated when 50% of fruit in a plot were ripe (30Wt). Yield in kg (YLD) was evaluated when 95% of the fruit in a plot were ripe. Ripe fruit were used to obtain the weight of 100 seeds (SW), which was measured only in 2009 due to labor limitations. The ripe fruit quality traits pH and Brix (*i.e.*, sugar content or soluble solids) were measured using a pureed sample of 10 whole fruit obtained from plots with 50% ripe fruit using a pH Testr2 (Oakton Instruments, Vernon Hills, IL) and a Reichert AR200 digital refractometer (Reichert Technologies, Buffalo, NY), respectively. Size traits obtained on ripe fruit were perimeter (FP), width (FW; width at mid-height), and height (FH; height at mid-width). These traits were measured on flatbed scanner images of eight longitudinally sliced fruit per plot using Tomato Analyzer software (Brewer *et al.* 2006), which refers to fruit length as height and fruit longitudinal circumference as perimeter. From FH and FW, the secondary variable fruit shape (FS; ratio of FH to FW) was obtained. Trait names, abbreviations, and brief descriptions are provided in Table 1.

**TABLE 1:** Abbreviations for Traits Evaluated in this Study

Trait Type	Abbreviation	Description
Late blight	LEAF	AUDPC for foliar symptoms
	STEM	AUDPC for stem symptoms
Maturity	DAP1st	Number of days after planting to first ripe fruit
	DAP50	Number of days after planting to 50% ripe fruit
Yield	YLD	Fruit yield (kg)
Fruit size/shape	FH	Fruit height (mm)
	FW	Fruit width (mm)
	FS	Fruit shape (FH×FW, mm^2^)
	FP	Fruit perimeter (mm)
	30Wt	Weight of 30 fruits (g)
Fruit quality	Brix	°Brix (soluble solids content)
	pH	Fruit acidity
Plant architecture	CD	Canopy density (visual rating, 1 = very sparse to 5 = very dense)
	HAB	Plant habit (visual rating, 1 = prostrate to 5 = very upright)
	H	Plant height (cm)
	W	Plant width (cm)
	SH	Plant shape (H:W, cm^2^)
	SZ	Plant size (H×W, cm^2^)
	SW	Weight of 100 seeds (g)

AUDPC, area under the disease progress curve.

On 15 September 2009, Salinas locations 1 and 2 were inoculated with a local *P. infestans* isolate as described in Johnson *et al.* (2012). In 2010 in mid-September, a natural *P. infestans* infection occurred in both locations, precluding the need for inoculation. As detailed in Johnson *et al.* (2012), phenotypic scoring of late blight disease symptoms was performed visually and symptom data were used to calculate area under the disease progress curve (AUDPC) for foliar and stem disease symptom progression (referred to as LEAF and STEM, respectively). Lower AUDPC values indicate less disease symptom progress and therefore are indicative of increased disease resistance.

### Statistical data analysis

Data for each trait (Table S3) was tested for normality using the Shapiro/Wilk W statistic in PROC UNIVARIATE in SAS version 9.1 (SAS Institute, Cary, NC) and for homogeneity of variance using Levene’s test. Data for heteroscedastic traits were weighted by the reciprocal of the variance for those terms with significant departure from the assumption of equal variance. ANOVA for each trait was performed using PROC GLM in SAS v.9.1 with the following linear additive model for a randomized complete block design and multiple locations:

$$\mathrm{Trait}=\mathrm{Loc}+\mathrm{Block}\left(\mathrm{Loc}\right)+\mathrm{Genotype}+{\mathrm{Loc}}^{*}\mathrm{Genotype}$$

where Trait was a given phenotypic trait, Loc was the effect of location, Genotype was the individual sub-NIL or control (NIL11, E6203, Hypeel 45), the * indicated an interaction, and parentheses indicated a nested variable. Block(Loc) was considered a random variable. Significant genotype × location interactions were detected in 2009 for CD, DAP1st, DAP50, FS, YLD, FW, and W, and in 2010 for CD, DAP50, FH, H, and SZ. For these traits, separate analyses were conducted for each location. PROC MIXED in SAS v.9.1 was used to estimate least squares means (LSMeans), due to missing data for some traits, and to perform means separation with Tukey’s HSD.

Pearson correlation coefficients (*r*) were calculated for pairwise combinations of all trait genotypic means in 2009 and in 2010 using Proc CORR in SAS v.9.1. Only significant (*P* ≤ 0.05) correlations ≥0.4 are reported.

### Linkage and QTL mapping

A linkage map for the chromosome 11 introgressed region was constructed using DNA marker genotype data across 21 loci for 852 BC_6_S_1_ progeny (150 recombinant, plus 702 nonrecombinant). The map was constructed with JoinMap 3.0 (Van Ooijen and Voorrips 2001) using the Kosambi mapping function and a 3-LOD significance threshold. We used a comparison of marker locations between our map and to the *S. lycopersicum* genome sequence (SL2.5; Sol Genomics Network, http://solgenomics.net) (Sato *et al.* 2012; Shearer *et al.* 2014) to estimate the physical size of the *S. lycopersicum* region replaced by the *S. habrochaites* introgression.

QTL mapping for each trait was performed with the composite interval mapping (CIM) module in WinQTLCartographer 2.5 (Wang *et al.* 2011) using sub-NIL means obtained from ANOVA for each trait. QTL mapping was performed using CIM Model 6 (Standard Model) and the forward and backward regression method with a walk speed of 1 cM and a window size of 2 cM. Trait-specific permuted LOD thresholds (*P* = 0.05) were empirically established for each trait using 1000 permutations (Churchill and Doerge 1994) in WinQTLCartographer.

A QTL for a trait was considered significant at *P* ≤ 0.05 if the peak LOD value exceeded the trait-specific permuted threshold. Multiple QTL were declared for a single trait when the LOD values between significant (*P* ≤ 0.05) peaks within the introgressed region decreased below the significance LOD threshold for at least two contiguous markers. Each significant QTL was denoted by trait name, location, and year. For example, DAP1st34_2009 is a QTL detected in the analysis of DAP1st data from locations 3 and 4 in 2009.

**Figure 1:**
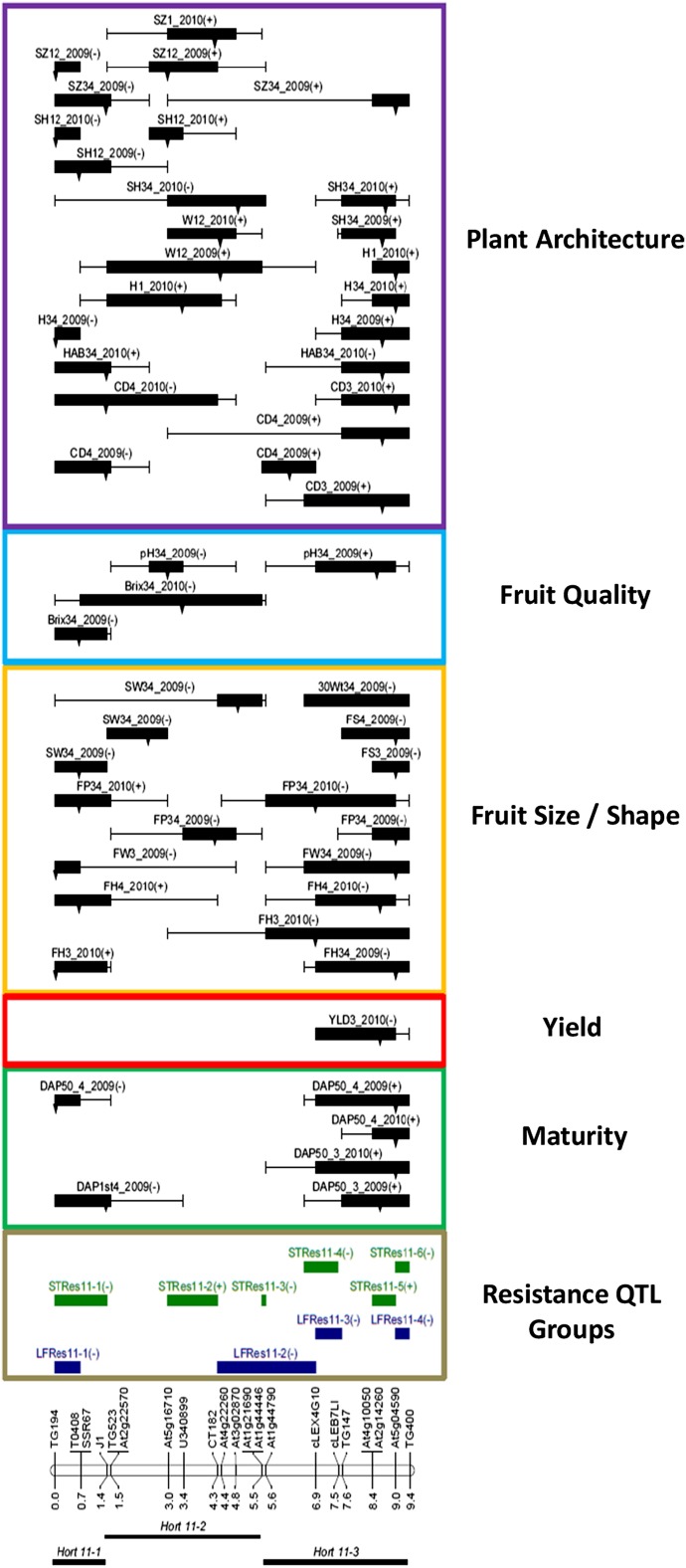
QTL mapped to a chromosome 11 region introgressed from *Solanum habrochaites* to *S. lycopersicum*. Horticultural trait QTL and *Phytophthora infestans* resistance QTL groups detected in chromosome 11 sub-NILs evaluated in 2009 and 2010 field experiments, sorted by trait class. Left of the linkage map are horticultural trait QTL group names, locations, and distances in cM; right of the linkage map are *P. infestans* resistance trait QTL groups (*LFRes* and *STRes* refer to LEAF and STEM resistance, respectively) (Johnson *et al.* 2012) and QTL detected for horticultural traits, sorted by trait class. Boxes and whiskers show 1-LOD and 2-LOD intervals, respectively. Arrows on QTL bars indicate LOD peak locations. QTL names are given by trait, location(s), and year evaluated (see *Materials and Methods*). The effect of the *S. habrochaites* allele at a QTL is indicated after the QTL name: (−) indicates a decrease in that trait value.

A linkage map figure showing locations of significant QTL was constructed using MapChart2.1 (Voorrips 2002). QTL locations were indicated as 1-LOD bars and 2-LOD whiskers (Figure 1). For easy reference and purposes of discussion, QTL were assigned to QTL trait groups (delineated as *Hort 11-1* through *Hort 11-3*) based on coincidence of their 1-LOD support intervals. Although a few of the QTL had 1-LOD support intervals that extended beyond the boundary of their assigned group, their peak locations supported their placement in these groups.

Comparisons were made among QTL for disease resistance traits (LEAF and STEM; Johnson *et al.* 2012) and horticultural traits for QTL coincidence by visual inspection of their chromosomal locations on the linkage map. A statistical test based on the hypergeometric probability distribution (Lin *et al.* 1995) was used to calculate QTL correspondence, the probability of obtaining the observed number of matching QTL by chance. A QTL match was declared when the one-LOD support intervals overlapped. The number of comparison intervals (n) was six, based on the average size of our QTL (1.84 cM) and the overall map distance of the introgression (9.4 cM).

Our QTL locations were also compared with those previously reported for both disease resistance and horticultural traits on chromosome 11 in tomato and in potato, based on common markers as well as genomic sequence data for both crop species. Sources used for QTL location comparisons included the following: tomato (Bernacchi *et al.* 1998b; Grandillo and Tanksley 1996; Semel *et al.* 2006); potato (Danan *et al.* 2011; Leonards-Schippers *et al.* 1994); and genomic sequences (http://solgenomics.net) (Sato *et al.* 2012; Xu *et al.* 2011). When common markers were not available, the Tomato-Expen 2000 map (Fulton *et al.* 2002) available on the Sol Genomics Network (http://solgenomics.net) (Bombarely *et al.* 2011) was used to facilitate map alignment.

### Selection of sub-NIL breeding lines

Truncation selection was applied sequentially for LEAF, YLD, FP, and 30Wt to identify breeding lines potentially useful for development of tomato varieties with improved resistance to *P. infestans*. Out of 42 sub-NILs, the first round of truncation removed 9 lines with leaf resistance scores below that of E6203 in 2 years or locations. The second round removed 2 lines with YLD <66% of E6203 in 2 years or locations, whereas the third round removed 7 lines with FP <92% of E6203 in 2 years or locations. The final round removed 2 lines with 30Wt <80% of E6203 in 2 years or locations. Maturity was also considered; however, two lines with significantly later maturity than E6203 in at least 1 year or location (08GH4106 and 08GH8032) were selected due to their relatively high levels of foliar resistance to *P. infestans* (*i.e.*, lower LEAF values). At the end of the process, 11 lines were chosen.

## Results

### ANOVAs

In 2009, 65 genotypes (sub-NILs and controls) were evaluated for late blight disease symptom traits (Johnson *et al.* 2012) and horticultural traits (Table 1). For all traits, genotypes were significantly different (*P* ≤ 0.05, Table 2). Significant genotype × location interactions were detected in 2009 for CD, DAP1st, DAP50, FS, YLD, FW, and W. As a result, each of these traits was analyzed separately by location. R^2^ values per trait ranged from 0.45 to 0.89.

**TABLE 2:** Summary of Analyses of Variance Performed on Trait Data

					F Values		
Trait Class	Trait Code	Trait	Year	Location	Genotype	Location	*R*^2^
Late blight resistance	LEAF	Leaf AUDPC	2009	1 & 2	2.28‡	16.71*	0.76
			2010	1 & 2	6.64‡	63.68‡	0.74
	STEM	Stem AUDPC	2009	1 & 2	9.37‡	1.30 ns	0.55
			2010	1	2.48*	—	0.52
				2	0.16 ns	—	0.62
Maturity	DAP1st	Days to 1st ripe fruit	2009	3	1.88†	—	0.49
				4	4.52‡	—	0.69
			2010	3 & 4	7.93‡	135.68^‡^	0.67
	DAP50	Days to 50% ripe fruit	2009	3	7.57‡	—	0.79
				4	4.74‡	—	0.71
			2010	3	12.40‡	—	0.81
				4	7.91‡	—	0.72
Yield	YLD	Yield	2009	3	1.91‡	—	0.48
				4	1.60*	—	0.45
			2010	3	2.84‡	—	0.48
Fruit size/shape	FH	Fruit height	2009	3 & 4	15.44‡	0.04 ns	0.80
			2010	3	18.78‡	—	0.86
				4	15.12‡	—	0.83
	FW	Fruit width	2009	3	5.56‡	—	0.73
				4	4.45‡	—	0.69
			2010	3 & 4	10.39‡	0.09 ns	0.65
	FS	Fruit shape	2009	3	17.45‡	—	0.89
				4	14.32‡	—	0.87
			2010	3 & 4	45.16‡	2.42 ns	0.88
	FP	Fruit size	2009	3 & 4	6.32‡	3.19 ns	0.66
			2010	3 & 4	14.70‡	0.19 ns	0.72
	30Wt	Fruit weight	2009	3 & 4	11.39‡	0.50 ns	0.76
			2010	3 & 4	24.41‡	3.64 ns	0.81
	SW	Seed weight	2009	3 & 4	15.95‡	1.69 ns	0.80
Fruit quality	Brix	^o^Brix	2009	3 & 4	8.39‡	13.87*	0.67
			2010	3 & 4	7.62‡	70.33‡	0.63
	pH	pH	2009	3 & 4	1.38*	12.17*	0.45
			2010	3 & 4	3.45‡	6.18*	0.45
Plant architecture	CD	Canopy density	2009	1 & 2	3.10‡	0.22 ns	0.50
				3	1.89†	—	0.50
				4	4.76‡	—	0.69
			2010	3	3.89‡	—	0.56
				4	2.86‡	—	0.49
	HAB	Plant habit	2009	1, 2, 3, 4	3.62‡	40.93‡	0.55
			2010	3 & 4	10.91‡	2.93 ns	0.66
	H	Plant height	2009	1 & 2	6.41‡	0.01 ns	0.66
				3 & 4	17.27‡	95.46‡	0.84
			2010	1	4.87‡	—	0.53
				2	1.76†	—	0.30
				3 & 4	10.52‡	0.66 ns	0.65
	W	Plant width	2009	1 & 2	4.16‡	1.01 ns	0.61
				3	2.71‡	—	0.57
				4	3.09‡	—	0.61
			2010	3 & 4	17.71‡	6.40*	0.77
				1 & 2	6.22‡	37.9‡	0.52
	SH	Plant shape	2009	1 & 2	2.70‡	1.01 ns	0.53
				3 & 4	1.74†	1.46 ns	0.66
			2010	1 & 2	9.45‡	102.23‡	0.60
				3 & 4	13.05‡	12.88*	0.71
	SZ	Plant size	2009	1 & 2	6.79‡	0.65 ns	0.68
				3 & 4	11.72‡	112.91‡	0.82
			2010	1	4.57‡	—	0.52
				2	1.32 ns	—	0.25
				3 & 4	17.14‡	2.24 ns	0.76

F test values and *R*^2^ values are presented for each analysis by trait, year, and location or combination of locations (see *Materials and Methods*). *R*^2^ indicates the fit of the data to the linear additive model for each analysis. Late blight disease resistance results are from Johnson *et al.* (2012). AUDPC, area under the disease progress curve; —, not included in model; ns, not significant.

* *P* ≤ 0.05

† *P* ≤ 0.01

‡ *P* ≤ 0.001

In 2010, 45 genotypes were analyzed for disease symptom traits and horticultural traits (Table 1). For all traits except SZ in location 2, genotypes were significantly different (*P* ≤ 0.05, Table 2). Significant genotype × location interactions were detected in 2010 for CD, DAP50, FH, H, and SZ. Therefore, these traits were analyzed separately by location. R^2^ values per trait ranged from 0.25 to 0.88. On average, foliar resistance to *P. infestans* (LEAF) had higher R^2^ values than stem resistance (STEM). Horticultural traits involved with fruit size measurements and maturity had higher R^2^ values than those involved with plant architecture.

### Means separation

There were significant (*P* ≤ 0.05) differences among genotype means for all traits, except for fruit pH in 2009 (Table S1). In general, sub-NILs with *S. habrochaites* introgressions at the marker loci At5g04590 and TG400 were significantly later maturing (DAP1st and/or DAP50) than control cultivar E6203 in at least one trait and year or location combination; however, some lines were exceptions to this trend. Most sub-NILs with *S. habrochaites* introgressions at the marker loci At5g04590 and TG400 also had significantly reduced FH compared with E6203. Relative to E6203, NIL11 exhibited significantly (*P* ≤ 0.05) greater foliar resistance to *P. infestans* (*i.e.*, lower LEAF values), but only in 2010. NIL11 also had later maturity (DAP50), reduced FH (in 2010 only), FP, 30Wt, SW, and W, increased H (Salinas locations only) and pH (in 2010 only), taller, narrower SH, and more prostrate habit than E6203. Sub-NILs 08GH3723, 08GH3999, 08GH4018, 08GH4106, and 08GH8032 displayed significantly (*P* ≤ 0.05) greater foliar resistance (LEAF) than E6203 in 2010; however, only 08GH8032 performed significantly better than E6203 in 2009. 08GH4228 had significantly better stem resistance (STEM) than E6203, but only in one location in 2010. None of the lines with significantly greater resistance showed any significant decrease in YLD or BRIX; however, several of them had significantly delayed maturity (DAP1st and DAP50), reduced fruit size (FH, FW, and FP) and 30Wt, and more upright HAB.

### Trait correlations

Pearson correlation coefficients (*r*) were obtained for *P. infestans* resistance trait means with horticultural trait means within each year (Table 3). Only significant correlations (*P* ≤ 0.05) ≥0.4 are discussed here. LEAF was moderately negatively correlated with CD at both Davis locations in 2009 (range: *r* = −0.49 to −0.51), but only in Davis location 3 in 2010 (*r* = −0.50). LEAF was also moderately negatively correlated with DAP1st in 2009 and 2010 (range: *r* = −0.44 to −0.57, and −0.46, respectively) and with DAP50 in 2009 (range: *r* = −0.48 to −0.62). STEM was moderately positively correlated with H in 2010, although the correlation was higher between some trait/location combinations than between others (range: *r* = 0.47–0.60). Significant (*P* ≤ 0.05) correlations were also found between pairs of horticultural traits (Table S2). Of particular note, CD was moderately positively correlated with maturity traits in both years (DAP1st *r* values 0.54–0.75; DAP50 *r* values 0.61–0.76), and FP was moderately negatively correlated with maturity traits DAP1st and DAP50 in both years (*r* values −0.53 to −0.65).

**TABLE 3:** Trait Correlations

**2009**	**LEAF12**		
DAP1st3	−0.44‡		
DAP1st4	−0.57‡		
DAP50_3	−0.48‡		
DAP50_4	−0.62‡		
FH34	0.55‡		
FP34	0.42‡		
CD3	−0.51‡		
CD4	−0.49‡		
H34	−0.61‡		

**2010**	**LEAF12**	**STEM1**	**STEM2**
DAP1st34	−0.46†		
CD3	−0.50‡		
HAB34	0.54‡		
H1		0.58‡	0.60‡
H34		0.47†	0.48†
W34			0.57‡

Pearson correlation coefficients (*r*) among *Phytophthora infestans* resistance traits (LEAF and STEM, data from Johnson *et al.* 2012) and horticultural traits were performed using genotype means. Only significant correlations ≥ 0.4 are presented. Trait names are given by year according to trait and location(s) (see *Materials & Methods*).

* *P* ≤ 0.05

† *P* ≤ 0.01

‡ *P* ≤ 0.001

### Linkage map

**Figure 2:**
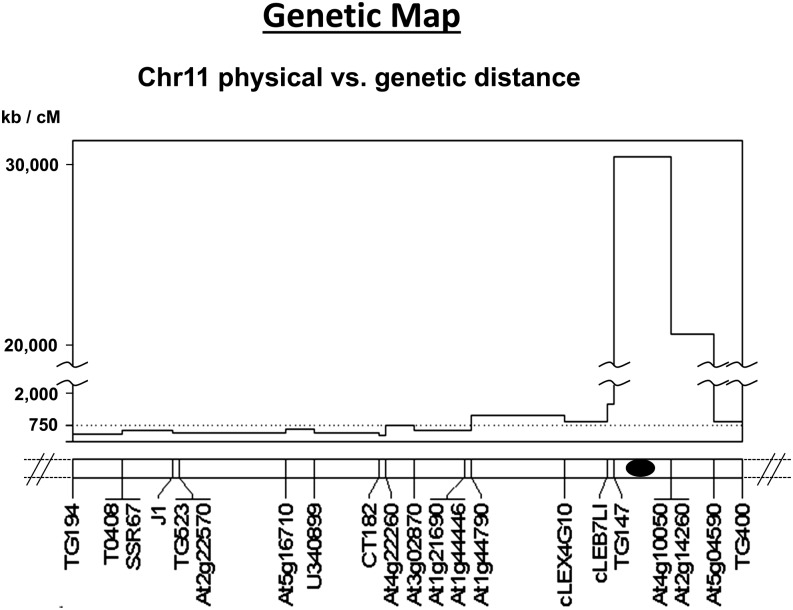
Estimated physical distance (based on the *S. lycopersicum* reference genome v2.50) *vs.* genetic distance in a chromosome 11 region introgressed from *S. habrochaites*. Vertical axis indicates the estimated ratio of physical distance to genetic distance for each marker interval on the linkage map for this region. Black oval indicates approximate centromere position.

The linkage map for the chromosome 11 introgressed region from *S. habrochaites* was 9.4 cM and spanned markers TG194 to TG400 (Figure 1). The average marker spacing was 0.5 cM and the largest gaps were each 1.5 cM in length, with one between markers At2g22570 and At5g16710 and another between At1g44790 and cLEX4G10. Comparison of our genetic map with the SL2.5 *S. lycopersicum* tomato genome sequence build (http://solgenomics.net) allowed estimation of the physical extent of *S. lycopersicum* DNA displaced by the *S. habrochaites* introgression. The 9.4-cM region from TG194 to TG400 corresponded to a physical distance of approximately 48.6 Mbp in *S. lycopersicum* and spans the centromere (Figure 2). With a mean of 5.17 Mbp per cM, this is 6.9-times higher than the genome-wide average ratio of genetic distance to physical distance, 750 kb per cM (Tanksley *et al.* 1992). The most intense recombination suppression occurred in the pericentromeric region between TG147 and At2g14260, where 1 cM corresponded to approximately 31.3 Mbp. Conversely, other parts of the chromosome 11 introgressed region from *S. habrochaites* exhibited slightly increased recombination relative to the genome-wide average (Figure 2). According to the ITAG 2.4 annotation of tomato chromosome 11, the interval from TG194 to TG400 contains 1473 genes. However, because a *S. habrochaites* genome sequence is not publicly available, the physical size and gene space of the introgression cannot be determined currently.

### Mapped QTL

Within the introgressed chromosome 11 region containing resistance QTL *lb11* (Brouwer and St. Clair 2004), we detected in this study 53 significant (*P* ≤ 0.05) QTL for 17 horticultural traits (Figure 1 and Table 4). In 2009, 30 QTL were detected; 23 QTL were detected in 2010. If we consider multiple coincident QTL for the same trait as a single, unique QTL, then a total of 34 unique QTL were mapped across the 17 traits.

**TABLE 4:** Summary of Significant QTL for Horticultural Traits

Trait Class	Trait Code	Trait	Group	Year	Location(s)	Peak Marker or Interval	Peak LOD/ Threshold LOD	*R*^2^
Maturity	DAP1st	Days to 1st ripe fruit	*Hort11-1*	2009	4	J1	1.81/1.66	0.09
	DAP50	Days to 50% ripe fruit	*Hort11-1*	2009	4	TG194	4.28/1.68	0.27
			*Hort11-3*	2009	3	At4g10050	3.65/1.74	0.24
					4	At5g04590	5.94/1.68	0.35
				2010	3	At5g04590	2.12/1.53	0.20
					4	At5g04590	3.67/1.76	0.33
Yield	YLD	Yield	*Hort11-3*	2010	3	At4g10050	4.14/1.69	0.35
Fruit size /shape	FH	Fruit height	*Hort11-1*	2010	3	TG194	4.56/1.69	0.38
					4	T0408	3.24/1.75	0.23
			*Hort11-3*	2009	3 & 4	At5g04590	4.78/1.64	0.20
			*Hort11-3*	2010	3	cLEX4G10	1.85/1.69	0.11
					4	At4g10050	3.61/1.75	0.32
	FW	Fruit width	*Hort11-1*	2009	3	TG194	2.10/1.75	0.12
	FS	Fruit shape	*Hort11-3*	2009	3	At5g04590	9.68/1.88	0.53
					4	At5g04590	7.01/1.69	0.41
	FP	Fruit size	*Hort11-1*	2010	3 & 4	T0408	2.40/1.65	0.18
			*Hort11-2*	2009	3 & 4	CT182	1.88/1.76	0.09
			*Hort11-3*	2009	3 & 4	At5g04590	3.16/1.76	0.15
				2010	3 & 4	cLEX4G10	1.74/1.65	0.13
	30Wt	Fruit weight	*Hort11-3*	2009	3 & 4	At5g04590	1.94/1.66	0.13
	SW	Seed weight	*Hort11-1*	2009	3 & 4	T0408	8.02/1.68	0.33
			*Hort11-2*	2009	3 & 4	At2g22570-At5g16710	8.17/1.68	0.48
			*Hort11-2*		3 & 4	At3g02870	2.70/1.68	0.09
Fruit quality	Brix	^o^Brix	*Hort11-1*	2009	3 & 4	T0408	3.17/1.76	0.21
				2010	3 & 4	U340899	1.98/1.70	0.19
	pH	pH	*Hort11-2*	2009	3 & 4	At5g16710	2.07/1.74	0.10
			*Hort11-3*	2009	3 & 4	At4g10050	4.98/1.74	0.29
Plant architecture	CD	Canopy density	*Hort11-1*	2009	4	J1	2.17/1.82	0.09
				2010	4	J1	1.88/1.67	0.18
			*Hort11-3*	2009	3	At4g10050	2.15/1.60	0.11
					4	At4g10050	1.96/1.82	0.09
					4	At1g44790-cLEX4G10	2.71/1.82	0.15
				2010	3	At5g04590	3.25/1.79	0.29
	HAB	Plant habit	*Hort11-1*	2010	3 & 4	J1	3.92/1.78	0.22
			*Hort11-3*	2010	3 & 4	At4g10050	3.09/1.78	0.15
	H	Plant height	*Hort11-1*	2009	3 & 4	TG194	9.11/1.74	0.48
			*Hort11-2*	2010	1	U340899	4.81/1.77	0.40
			*Hort11-3*	2009	3 & 4	At4g10050	5.95/1.74	0.21
				2010	1	At5g04590	2.56/1.77	0.19
					3 & 4	At5g04590	2.93/1.73	0.27
	W	Plant width	*Hort11-2*	2009	1 & 2	At4g22260	1.96/1.77	0.13
				2010	1 & 2	At4g22260	2.52/1.59	0.23
	SH	Plant shape	*Hort11-1*	2009	1 & 2	T0408	3.10/1.71	0.21
				2010	1 & 2	TG194	3.63/1.48	0.32
			*Hort11-2*	2010	1 & 2	At5g16710	1.65/1.48	0.13
					3 & 4	At3g02870	1.93/1.72	0.13
			*Hort11-3*	2009	3 & 4	At4g10050	4.68/1.58	0.21
				2010	3 & 4	At4g10050	4.57/1.72	0.36
	SZ	Plant size	*Hort11-1*	2009	1 & 2	TG194	2.27/1.68	0.14
					3 & 4	J1	2.28/1.69	0.09
			*Hort11-2*	2009	1 & 2	At5g16710	1.77/1.68	0.11
				2010	1	CT182	3.32/1.76	0.30
			*Hort11-3*	2009	3 & 4	At5g04590	2.08/1.69	0.08

Trait Class	Trait Code	Trait	Group	Year	Location(s)	Allele Direction	1-LOD Support Interval	Flanking Markers
Maturity	DAP1st	Days to 1st ripe fruit	*Hort11-1*	2009	4	(‒)	0.0‒1.5	TG194-TG523
	DAP50	Days to 50% ripe fruit	*Hort11-1*	2009	4	(‒)	0.0‒0.7	TG194-T0408
			*Hort11-3*	2009	3	(+)	7.6‒9.4	TG147-TG400
					4	(+)	6.9‒9.4	cLEX4G10-TG400
				2010	3	(+)	6.9‒9.4	cLEX4G10-TG400
					4	(+)	8.4‒9.4	At2g14260-TG400
Yield	YLD	Yield	*Hort11-3*	2010	3	(‒)	6.9‒9.0	cLEX4G10-At5g04590
Fruit size/shape	FH	Fruit height	*Hort11-1*	2010	3	(+)	0.0‒1.4	TG194-J1
					4	(+)	0.0‒1.5	TG194-TG523
			*Hort11-3*	2009	3 & 4	(‒)	6.9‒9.4	cLEX4G10-TG400
				2010	3	(‒)	5.6‒9.4	At1g44790-TG400
					4	(‒)	6.9‒9.0	cLEX4G10-At5g04590
	FW	Fruit width	*Hort11-1*	2009	3	(‒)	0.0‒0.7	TG194-T0408
	FS	Fruit shape	*Hort11-3*	2009	3	(‒)	8.4‒9.4	At2g14260-TG400
					4	(‒)	7.6‒9.4	TG147-TG400
	FP	Fruit size	*Hort11-1*	2010	3 & 4	(+)	0.0‒1.5	TG194-TG523
			*Hort11-2*	2009	3 & 4	(‒)	3.4‒4.8	U340899-At3g02870
			*Hort11-3*	2009	3 & 4	(‒)	8.4‒9.4	At2g14260-TG400
				2010	3 & 4	(‒)	5.6‒9.0	At1g44790-At5g04590
	30Wt	Fruit weight	*Hort11-3*	2009	3 & 4	(‒)	6.6‒9.4	At1g44790-TG400
	SW	Seed weight	*Hort11-1*	2009	3 & 4	(‒)	0.0‒1.4	TG194-J1
			*Hort11-2*	2009	3 & 4	(‒)	1.4‒3.0	J1-At5g16710
					3 & 4	(‒)	4.3‒5.5	CT182-At1g21690
Fruit quality	Brix	^o^Brix	*Hort11-1*	2009	3 & 4	(‒)	0.0‒1.4	TG194-J1
				2010	3 & 4	(‒)	0.7‒5.5	SSR67-AT1g21690
	pH	pH	*Hort11-2*	2009	3 & 4	(‒)	2.5‒3.4	At2g22570-U340899
			*Hort11-3*	2009	3 & 4	(+)	6.9‒9.0	cLEX4G10-At5g04590
Plant architecture	CD	Canopy density	*Hort11-1*	2009	4	(‒)	0.0‒1.5	TG194-TG523
				2010	4	(‒)	0.0‒4.3	TG194-CT182
			*Hort11-3*	2009	3	(+)	6.6‒9.4	At1g44790-TG400
					4	(+)	7.6‒9.4	TG147-TG400
					4	(+)	5.5‒6.9	At1g21690-cLEX4G10
				2010	3	(+)	7.6‒9.4	TG147-TG400
	HAB	Plant habit	*Hort11-1*	2010	3 & 4	(+)	0.0‒1.5	TG194-TG523
			*Hort11-3*	2010	3 & 4	(‒)	7.6‒9.4	TG147-TG400
	H	Plant height	*Hort11-1*	2009	3 & 4	(‒)	0.0‒0.7	TG194-T0408
			*Hort11-2*	2010	1	(+)	1.4‒4.4	J1-At4g22260
			*Hort11-3*	2009	3 & 4	(+)	7.6‒9.4	TG147-TG400
				2010	1	(+)	8.4‒9.4	At2g14260-TG400
					3 & 4	(+)	8.4‒9.4	At2g14260-TG400
	W	Plant width	*Hort11-2*	2009	1 & 2	(+)	1.4‒5.5	J1-At1g21690
				2010	1 & 2	(+)	3.0‒4.8	At5g16710-At3g02870
	SH	Plant shape	*Hort11-1*	2009	1 & 2	(‒)	0.0‒1.5	TG194-TG523
				2010	1 & 2	(‒)	0.0‒0.7	TG194-T0408
			*Hort11-2*	2010	1 & 2	(+)	2.5‒3.4	At2g22570-U340899
					3 & 4	(‒)	3.0‒5.6	At5g16710-At1g44790
			*Hort11-3*	2009	3 & 4	(+)	7.6‒9.0	TG147-At5g04590
				2010	3 & 4	(+)	7.6‒9.0	TG147-At5g04590
	SZ	Plant size	*Hort11-1*	2009	1 & 2	(‒)	0.0‒0.7	TG194-T0408
					3 & 4	(‒)	0.0‒1.5	TG194-TG523
			*Hort11-2*	2009	1 & 2	(+)	2.5‒4.3	At2g22570-CT182
				2010	1	(+)	3.0‒4.8	At5g16710-At3g02870
			*Hort11-3*	2009	3 & 4	(+)	8.4‒9.4	At2g14260-TG400

Group indicates coincident QTL, as defined by colocation of the 1-LOD intervals. *R*^2^ values are the proportion of phenotypic variation explained by the marker-trait association. Allele direction is the direction of the effect of the *S. habrochaites* allele at that QTL, in terms of the trait being measured. The 1-LOD support interval positions refer to the cM distances on the linkage map for the introgressed region from *S. habrochaites*. See Johnson *et al.* (2012) for LEAF and STEM QTL results.

### Horticultural trait QTL groups

Based on their location on the linkage map, three major QTL groups (*Hort11-1* through *Hort11-3*) were delineated (Figure 1 and Table 4) as described in *Materials and Methods*. *Hort11-1* contained QTL for maturity (DAP1st and DAP50), plant architecture (CD, HAB, H, SH, and SZ), fruit size (FH, FW, and FP), fruit weight, seed weight, and Brix (Figure 1 and Table 4). Plants with the wild allele at these QTL had reduced plant size and height, were more upright, and had a dense plant canopy. The wild allele at these QTL reduced fruit weight and Brix only in 2009, and increased fruit size and fruit height in 2010. The number of days to maturity was also significantly reduced by the wild allele at *Hort11-1*, but only in one location in 2009.

The *Hort11-2* QTL group contained QTL for plant architecture (CD, H, W, SH, and SZ), fruit size, seed weight, and pH (Figure 1 and Table 4). The *S. habrochaites* allele at these QTL resulted in slightly larger (both taller and wider) plants. There was evidence of genotype × environment interaction (G × E) for plant shape, because the presence of the wild allele caused a taller, narrower phenotype at the Salinas locations and a shorter, wider phenotype in Davis. Plants with the wild allele at these QTL also had smaller fruit with lower seed weight and lower pH. The *Hort11-3* QTL group contained QTL for yield, maturity (DAP50 only), plant architecture (CD, HAB, H, SH, and SZ), fruit shape, fruit size (FH, FW, and FP), and pH (Table 4). Plants with the wild allele at these QTL had increased canopy density and plant height, but they also had a more prostrate plant habit. The wild allele at these QTL also reduced fruit height, weight, and yield, slightly increased fruit pH, and slightly delayed maturity.

Plant architecture was controlled by the *S. habrochaites* introgression at each of the three horticultural QTL groups. *Hort11-1* produced smaller, shorter plants; *Hort11-3* produced larger, taller plants; and *Hort11-2* affected plant size differently depending on the environment in which the plants were grown. The number of days to maturity was reduced by *Hort11-1* but was increased by *Hort11-3*. Yield was only affected by *Hort11-3*, and only in a single environment. Fruit size was increased by *Hort11-1* and reduced by *Hort11-2* and *Hort11-3*, whereas fruit shape was only affected by *Hort11-3*. Brix was reduced by *Hort11-1* and *Hort11-2*. Fruit pH was reduced by *Hort11-2* and increased by *Hort11-3*.

### Horticulture trait QTL and linkage with *P. infestans* resistance QTL

In our companion study by Johnson *et al.* (2012), we detected four and six QTL groups within the introgressed chromosome 11 region controlling foliar (LEAF) and stem (STEM) resistance to *P. infestans*, respectively, with the QTL groups designated as *LFRes11-1* through *LFRes11-4* and *STRes11-1* through *STRes11-6* (Figure 1) (Johnson *et al.* 2012). We used markers in common to align the QTL groups by visual inspection. *LFRes11-1* and *STRes11-1* were collocated with *Hort11-1* (Figure 1). *LFRes11-2*, *STRes11-2*, and *STRes11-3* were co-located with *Hort11-2*. *LFRes11-3*, *LFRes11-4*, *STRes11-4*, *STRes11-5*, *and STRes11-6* were co-located with *Hort11-3*.

The *Hort11-1* QTL group included QTL for 12 of the 17 horticultural traits measured. Most of the horticultural trait QTL within this group mapped within marker interval TG194-J1 (Figure 1 and Table 4). *LFRes11-1* was a single QTL spanning the interval TG194-T0408, whereas *STRes11-1* consisted of a pair of QTL spanning the interval TG194-T0408 and TG194-J1. All three of these resistance QTL had LOD peaks at TG194. Other than the QTL for DAP50, FH, FW, H, SH, and SZ, the horticultural trait QTL in *Hort11-1* had LOD peaks at loci other than TG194, suggesting that many of these QTL may be only linked to the resistance loci, rather than being pleiotropic effects of the resistance QTL in *LFRes11-1* and *STRes11-1*.

QTL were detected in the *Hort11-2* group for 8 of the 17 traits measured within the interval J1–At1g21690 (Figure 1 and Table 4). This interval contains *STRes11-2*, a set of three QTL spanning the interval At5g16710-CT182, two with LOD peaks at At5g16710 and one at U340899. *LFRes11-2* consisted of three resistance QTL, one of which spanned the interval CT182–At1g21690 with its LOD peak at At4g22260 located completely within the *Hort11-2* region. Another resistance QTL spanned the interval At3g02870–At1g44790, stretching to the end of *Hort11-2* and to the beginning of *Hort11-3* at At1g44790. This resistance QTL is only co-located with the *Hort11-2* QTL for SW, W, and SH. The third resistance QTL in *LFRes11-2* spanned At1g21690–cLEX4G10, falling mostly within the *Hort11-3* region. The last two *LFRes11-2* QTL both had peaks at At1g21690, suggesting the possibility of multiple resistance loci within this group. Also, within the border between *Hort11*-2 and *Hort11-3* is *STRes11-3*, spanning the interval At1g21690–At1g44790 and consisting of a single QTL with its peak at At1g21690.

The *Hort11-3* QTL group included QTL for 11 of the 17 horticultural traits evaluated within the interval At1g44790–TG400 (Figure 1 and Table 4). The portion of this group that overlaps *LFRes11-2* contains QTL where the *S. habrochaites* allele reduces FH, FP, and increases CD. The *Hort11-3* interval also contains *STRes11-4*, a single QTL flanked by the markers AT1g44790 and cLEB7LI, and *LFRes11-3*, a group of three QTL flanked by the markers cLEX4G10 and TG147. Within the portion of *Hort11-3* that corresponded to these resistance QTL groups, there were QTL for which the *S. habrochaites* allele reduced FH and FP. Although the 1-LOD intervals of QTL for maturity, YLD, FW, pH, and CD also overlapped these resistance QTL intervals, their peak locations suggested that the loci controlling these phenotypes are relatively distantly linked to those conferring resistance. Another resistance QTL group within the *Hort11-3* interval, *STRes11-5* was a single QTL in the interval At4g10050–At5g04590, with its peak at At5g04590, where the *S. lycopersicum* allele conferred resistance to *P. infestans*. Within this interval are QTL in *Hort11-3* where the presence of the *S. habrochaites* allele delayed maturity (DAP50), reduced YLD, FH, FW, FP, and FS, increased pH, CD, H, SH, and SZ, and conferred a more prostrate HAB. The last two resistance QTL groups within the *Hort11-3* interval, *LFRes11-4* and *STRes11-6*, consisted of four and two QTL, respectively, each within the interval At5g04590–TG400 and each having peak LOD at At5g04590. Within this interval are QTL from *Hort11-3*, where the *S. habrochaites* allele delayed maturity (DAP50), reduced FH, FW, FP, and FS, increased CD, H, and SZ, and conferred a more prostrate habit.

### QTL stability and QTL × environment interaction

None of the QTL in *Hort 11-1* was detected in all years and locations. The fruit size (FH and FP) QTL in this group were identified at both Davis locations, but only in 2010. Similarly, the Brix QTL was mapped only in 2009. The maturity QTL in this group were only detected in a single year and location, suggesting an environmental influence on QTL expression.

Similarly, none of the QTL in *Hort11-2* was identified in all years and locations. The two QTL for SW were detected in both locations; however, data were only collected for this trait in 2009, so their stability over the years is unknown. Other QTL, such as those affecting Brix, pH, and FP were mapped in both locations, but only in a single year. QTL for plant size (W and SZ) were detected over both years, but only at the two Salinas locations in 2009 and only at a single location in 2010. Their effect may have been accentuated by the narrow row spacing, relative to that at the Davis locations. QTL for SH were detected in this group with opposite effect depending on location, another manifestation of QTL × E. In 2010, the *S. habrochaites* allele conferred a shorter, wider phenotype at the Davis locations, but a taller, narrower phenotype at the Salinas locations. This may also be due to the differences in row spacing between the two pairs of experiments. The difference in LOD peak location and only minor overlap between these two QTL may also indicate that these are separate, linked loci of opposite effects that are most pronounced in contrasting environments.

In the *Hort11-3* QTL group, the DAP50, FH, and FP QTL appear to be stable, being identified in each year and location. However, the LOD peaks for the FH and FP QTL over the 2 yr are sufficiently far apart to suggest that there may be multiple linked loci contributing to the effects for each trait. QTL for H were mapped at both Davis locations in both years but only at one of the Salinas locations, and only in 2010. QTL for CD were also relatively stable, being detected in three of the four year/location combinations; however, an additional CD QTL was found at location 4 in 2009, distal to those three QTL. QTL for FW, FS, and pH were identified in both locations in 2009, but not in 2010. The HAB QTL was mapped only in the Davis locations in 2010 and was not detected in 2009. The SZ QTL was only found at the Davis locations and only in 2009. The QTL for YLD was only detected in a single location and year.

### Coincidence of QTL between horticultural and resistance traits

The hypergeometric probability distribution was used to assess the significance of correspondence of QTL for *P. infestans* resistance with those for horticultural traits. No significant correspondence was detected between resistance (LEAF or STEM) and any of the horticultural traits evaluated in this study.

### Selection of sub-NIL breeding lines

Eleven lines were selected as being potentially useful as breeding lines for the development of tomato varieties with higher resistance to *P. infestans*: 08GH4106, 08GH3999, 08GH8032, 08GH3688, 08GH3765, 08GH4305, 08GH4794, 08GH4265, 08GH5362, and 08GH5285. We compared these lines with cultivar E6203 to evaluate their potential in breeding. 08GH3999 had significantly greater foliar resistance (*i.e.*, lower LEAF values) in both Salinas locations in 2010, but 08GH8032 was the only line that significantly outperformed E6203 in all Salinas locations and years. 08GH3765 had higher Brix in all years/locations, although only significantly so in 2010. Some of these selected lines also had undesirable horticultural traits, such as delayed maturity (08GH3765, 08GH4106, and 08GH8032), taller plant height (08GH4106 and 08GH5285 at the Davis locations), smaller FP for all years/locations (08GH4106 and 08GH8032), and lower 30Wt for all years/locations (08GH3999, 08GH4106, and 08GH8032).

## Discussion

### Genetic architecture of horticultural traits

A complex genetic architecture for traits, including maturity, fruit size and shape, soluble solids, seed weight, canopy density, and plant size and shape, was revealed by our mapping of this 9.4-cM introgressed region from *S. habrochaites* on tomato chromosome 11 at higher resolution (Figure 1 and Table 4). Fractionation of QTL, co-location or tight linkage of QTL for multiple traits, suppression of recombination near the centromere, and interaction between QTL and the experimental environment in which plants were grown each contributed to the complexity of the genetic architecture of this introgressed region.

Previously, Brouwer and St. Clair (2004) used NILs for the chromosome 11 region introgressed from *S. habrochaites* to fine-map late blight disease resistance and horticultural trait QTL for canopy density and fruit size within the marker interval TG194–TG400. The presence of wild alleles at these QTL resulted in a more open canopy and reduced fruit size, respectively. The NILs in that study were also evaluated for plant height, plant size, plant shape (referred to here as plant habit), maturity, and yield, but no significant QTL were detected for these traits.

In our present study, two QTL for canopy density mapped in this region, with the alleles conferring a more open canopy linked in repulsion phase. Three QTL for fruit size were detected, two linked in coupling phase with the wild allele reducing fruit size and the third linked in repulsion phase with the wild allele increasing fruit size. Closely linked QTL controlling a single trait have also been reported in other studies using interspecific crosses in tomato (Fridman *et al.* 2002; Johnson *et al.* 2012; Yates *et al.* 2004). Furthermore, our companion study that evaluated the same set of horticultural traits in a set of sub-NILs for a chromosome 5 region introgressed from *S. habrochaites* also discovered closely linked QTL for single traits (Haggard *et al.* 2013). Fractionation of QTL at higher-resolution mapping has also been observed in other studies (Chen and Tanksley 2004; Lecomte *et al.* 2004; Studer and Doebley 2011). In contrast, QTL for tomato soluble solids content (Brix), yield, fruit shape (Monforte and Tanksley 2000), and fruit weight (Alpert *et al.* 1995; Alpert and Tanksley 1996) were each resolved to a single locus with high-resolution mapping.

In addition to the fractionation of the horticultural trait QTL Brouwer and St. Clair (2004) had previously mapped in this region of chromosome 11, we also mapped QTL for plant height, plant size, plant habit, maturity, and yield. This result was unexpected, because Brouwer and St. Clair (2004) did not detect any significant QTL for these traits in this same region. With the exception of yield, we found that each of these traits was controlled by two or three QTL within the region of chromosome 11 introgressed from *S. habrochaites*, with the QTL alleles being linked in repulsion phase in relation to disease resistance QTL alleles. The opposite direction of effect of the *S. habrochaites* alleles at these QTL could have prevented their detection when combined in a single NIL, which may explain the results reported by Brouwer and St. Clair (2004).

Brouwer and St. Clair (2004) originally reported the association of *lb11b* with QTL controlling canopy density and fruit size. Johnson *et al.* (2012) described the fractionation of this chromosome 11 QTL for resistance to *P. infestans* into multiple QTL of smaller effect. In the present study, we used the same set of sub-NILs for chromosome 11 as Johnson *et al.* (2012) and mapped QTL for multiple horticultural traits that were also linked to *P. infestans* resistance QTL. Studies on the introgression of disease and pest resistance genes and QTL from wild species into their cultivated crop relatives have often reported linked horticultural trait QTL, referred to as linkage drag. Examples of interspecific linkage drag are found in tomato (Robert *et al.* 2001; Tanksley *et al.* 1998) and its related crop species potato (Danan *et al.* 2011; Visker *et al.* 2003). Linkage of horticultural trait QTL with disease/pest resistance QTL has also been reported in intraspecific populations of potato (Bradshaw *et al.* 2004), pepper (Barchi *et al.* 2007; Ben Chaim *et al.* 2001), bean (Ender and Kelly 2005; Miklas *et al.* 2003; Mkwaila *et al.* 2011), and cacao (Brown *et al.* 2007). Cosegregation of QTL for horticultural traits with genes or QTL for disease/pest resistance may be observed due to suppression of recombination between the loci controlling these traits. Recombination suppression is more likely in introgressions from wild species and in pericentromeric chromosomal regions such as that containing *lb11b* (see *Mapping in centromeric regions* below). Chromosomal inversions can also be a cause of repressed recombination, although we do not have any evidence to suggest the presence of inversions on chromosome 11 between cultivated and wild tomato. When we compared our map to the available interspecific linkage maps in tomato (SGN, http://solgenomics.net), marker orders were collinear and our map had one-third to one-fifth the amount of recombination in the interval where the majority of the suppression occurred. Because marker order was not rearranged among linkage maps, this suggests (but does not prove) an inversion is unlikely to be involved.

Another possible explanation for the association of horticultural traits with resistance QTL is that they may be related causally to the resistance. Factors such as maturity, plant height, lodging resistance, and canopy density can all contribute to avoidance of the consequences of environmental conditions favorable to pathogen infection, growth, and/or inoculum production.

### Tight linkage and pleiotropy

The co-location of QTL controlling multiple horticultural traits with each *P. infestans* resistance QTL (Figure 1) may be due to tight linkage and/or pleiotropy (Brown 2002; Chen and Luebberstedt 2010). In this study, we used recombinant sub-NILs for chromosome 11 to map QTL at higher resolution than in the study by Brouwer and St. Clair (2004), who reported QTL controlling resistance to *P. infestans* linked with QTL for canopy density and fruit size. Our results here suggest tight linkage between QTL groups *LFRes11-1*, *11-3*, and *11-4* controlling foliar resistance to *P. infestans* (LEAF) (Johnson *et al.* 2012) and QTL controlling maturity (Figure 1). This linkage is particularly interesting due to previously reported correlations between these traits and co-localization of maturity and resistance QTL in potato (see *P. infestans resistance and maturity traits* below). This result suggests that *LFRes11-1* and *11-2* may be especially desirable targets for selection (see *Implications for tomato breeding*, below) as they conferred resistance plus a positive effect and no effect on maturity, respectively.

Each of the three horticultural QTL groups on chromosome 11 was coincident with *P. infestans* resistance QTL. The linkage relationships between the co-located horticultural QTL and resistance QTL are not resolvable without identifying larger numbers of additional recombinants (Chen and Luebberstedt 2010; Mackay *et al.* 2009). Some horticultural QTL, however, had 1-LOD support intervals that did not overlap with resistance QTL groups. The maturity QTL in *Hort11-1* and *Hort11-3* did not overlap *LFRes11-2* and *STRes11-2* and *11-3*, and thus are likely to be closely linked, rather than pleiotropic, to the resistance QTL in these groups (Figure 1). Similarly, the *Hort 11-1* and *11-2* Brix QTL are likely to be closely linked, rather than pleiotropic, to *LFRes11-3* and *11-4* and to *STRes11-4*, *11-5*, and *11-6*. Also, the yield QTL in *Hort11-3* is also likely to be closely linked, not pleiotropic, to *LFRes11-1* and *11-2* and to *STRes11-1*, *11-2*, and *11-3*. Tight linkage was determined as the cause of coincidence among QTL controlling different traits in tomato studies that pursued higher resolution QTL mapping using sub-NILs (Lecomte *et al.* 2004; Monforte *et al.* 2001).

### Stability of QTL and QTL × environment interaction

In our study, the majority of QTL we discovered were stably expressed over environments and were detected at the same or very similar map position over multiple years and/or locations. Of the 34 horticultural trait QTL mapped, 29 were stably expressed over multiple environments, including QTL for maturity (DAP50 only), fruit size and shape, Brix, acidity, canopy density, plant habit, and plant size and shape. Our inferences regarding QTL stability are limited to only two locations over 2 years for all traits except plant architecture traits, which were evaluated all four locations (Salinas and Davis). The most stable QTL, those detected in all years and locations, were QTL for seed weight in *Hort11-1* and *Hort11-2*, and for fruit height and maturity (DAP50) in *Hort11-3*. Of the 34 QTL detected, only five were detected at a single location within a single year.

Interaction between a QTL and the environments in which it is evaluated (*e.g.*, years, locations, etc.) is known as QTL × E and may result in inconsistent detection of QTL across those environments (Bernardo 2008; Mackay *et al.* 2009; Xu and Crouch 2008). QTL with environmental interactions tend to be more difficult to utilize in breeding because they may have reduced selection efficiency. QTL × E may render an otherwise beneficial allele useless if it is not consistently expressed in the target environment (Xu and Crouch 2008). Traits that exhibited QTL × E in our study included maturity (DAP1st and DAP50), yield, fruit width, plant height, and plant size. Both stability and instability of QTL (*i.e.*, QTL × E) have been commonly reported in populations evaluated in multiple environments, including tomato (Bernacchi *et al.* 1998b; Frary *et al.* 2004; Haggard *et al.* 2013), potato (Constanzo *et al.* 2005), and maize (Peng *et al.* 2011).

Similar to our previous comparison with other tomato mapping studies, as described in Haggard *et al.* (2013), in this study we detected 14% of QTL only in a single environment, placing our observation within the middle range of other tomato mapping studies (Bernacchi *et al.* 1998b; Brouwer and St. Clair 2004; Do *et al.* 2010; Eshed *et al.* 1996; Eshed and Zamir 1996; Frary *et al.* 2004; Johnson *et al.* 2012; Tanksley *et al.* 1996). Although Haggard *et al.* (2013) also found QTL × E among mapped QTL for horticultural traits, the specific traits controlled by QTL exhibiting environmental interactions varied between the two studies. Whereas the present study found QTL × E effects for maturity (DAP1st and DAP50; *Hort11-1*) and yield QTL, Haggard *et al.* (2013) reported environmental stability for all QTL controlling these traits. All of the 17 horticultural traits that were evaluated exhibited QTL × E for at least one of their controlling loci across these two QTL mapping studies.

Changes in the direction of a QTL’s allelic effect from one location to the next may also be due to QTL × E. In this study, this was observed for QTL controlling plant shape in *Hort11-2*. In 2010, this QTL resulted in shorter, wider plants at both Davis locations, but taller, narrower plants at both Salinas locations. Other studies have reported changes in the direction of allelic effect depending on environment, for example, QTL controlling yield, fruit color, and Brix in tomato (Bernacchi *et al.* 1998a) yield in maize (Bouchez *et al.* 2002; Moreau *et al.* 2004) and plant height and kernel weight in wheat (Campbell *et al.* 2003). This phenomenon was also observed for plant architecture in our study of an introgression from *S. habrochaites* on tomato chromosome 5 (Haggard *et al.* 2013). Such changes in QTL allele directionality among locations or years may result from differences in environmental factors such as soil type, moisture, row spacing, soil fertility, sunlight intensity, or temperature.

### QTL comparisons with previously mapped QTL in tomato and potato

Some of the disease resistance and horticultural QTL reported here may correspond to those previously reported on chromosome 11 in tomato interspecific crosses. Although most previously reported QTL maps lack the resolution necessary for determining precise locations of QTL, most were sufficient for comparison based on common markers informed by genomic sequence data. Grandillo and Tanksley (1996) detected a QTL for thousand seed weight in a population derived from *S. lycopersicum* × *S. pimpinellifolium* that may correspond to our *Hort11-1* and/or *Hort11-2* SW QTL. In an advanced backcross population derived from *S. lycopersicum* × *S. habrochaites*, Bernacchi *et al.* (1998b) detected an environmentally influenced QTL for yield near the interval TG523–TG400, which may correspond to our *Hort11-3* YLD QTL detected only in a single location in 2010. They also mapped QTL for soluble solids content (Brix) in that marker interval that may correspond to our Brix QTL in *Hort11-1* and/or *Hort11-2*. Using a set of introgression lines (ILs) derived from a cross between *S. lycopersicum* and *S. pennellii*, Semel *et al.* (2006) discovered QTL for fruit length, fruit width, and seed weight in IL11-1 that may correspond to our *Hort11-1* QTL for FH, FW, and SW, respectively. Semel *et al.* (2006) also found QTL in IL11-2 for Brix, fruit width, and seed weight that may correspond to our *Hort11-1* QTL for Brix, FW, and SW, respectively. Alternatively, the Brix and seed weight QTL they identified may instead correspond to our *Hort11-2* QTL for Brix and SW. In IL11-3, Semel *et al.* (2006) detected QTL for fruit length and fruit width, which may correspond to our *Hort11-3* QTL for FH and FW, respectively.

QTL for resistance to *P. infestans* have also been reported on chromosome 11 in a meta-QTL analysis of potato (Danan *et al.* 2011), but few studies have reported linked QTL controlling horticultural traits analogous to those evaluated in the current study. This potato meta-QTL analysis study reported two QTL for *P. infestans* resistance linked to two QTL for maturity in the interval CP58A-GP250a, which appears to fall entirely within the chromosome 11 region we have investigated. Our finding of multiple linked QTL controlling these two traits within this region agrees with these results in potato.

### *P. infestans* resistance and maturity

Pearson correlation coefficients for foliar resistance to *P. infestans* (LEAF) and maturity (DAP1st and DAP50) were significantly negatively correlated (Table 3), suggesting that later maturity was associated with increased resistance. Three of the four LEAF QTL detected in this region of chromosome 11 were co-located with QTL for maturity traits (Figure 1). The results of a statistical test of QTL correspondence based on the hypergeometric probability function were not significant, however, suggesting that the co-location of QTL controlling these two traits may merely be due to chance. For *LFRes11-3* and *11-4*, our findings support the correlation of resistance with late maturity, because these QTL are co-located with the *Hort11-3* maturity QTL. However, it is noteworthy that *LFRes11-1* was co-located with QTL conferring earlier maturity in *Hort11-1*, and that *LFRes11-3* appears to only be closely linked to the maturity QTL in *Hort11-3*. This suggests that if delayed maturity is a component of the resistance conferred by the *S. habrochaites* allele at the *LFRes11-4* QTL, there may be different bases for the foliar resistance controlled by the other three QTL. Our parallel investigation of a chromosome 5 introgression from *S. habrochaites* also found significant correlation of *P. infestans* resistance and late maturity, along with maturity QTL co-located with each resistance QTL (Haggard *et al.* 2013).

Studies of *P. infestans* resistance in potato have also noted significant positive correlation between increased resistance and delayed maturity (Bradshaw *et al.* 2004; Collins *et al.* 1999; Danan *et al.* 2009; Toxopeus 1958). Alignment of our tomato chromosome 11 map with the potato MetaQTL map (Danan *et al.* 2011) using common markers, facilitated by the Tomato-Expen 2000 map (Fulton *et al.* 2002) on Sol Genomics Network (http://solgenomics.net), suggests that the potato QTL for resistance on chromosome 11 (*MQTL_1_late_blight*) may correspond to either *LFRes11-2* or *LFRes11-3*. The *MQTL_2_late_blight* potato resistance QTL and the *MQTL_1_maturity* and/or the *MQTL_2_maturity* QTL likely correspond to *LFRes11-4* and the *Hort11-3* maturity QTL, respectively.

### Mapping in centromeric regions

QTL mapping in heterochromatic centromeric regions is complicated by severe recombination suppression. It is likely that recombination suppression between TG147 and At5g04590, due to their centromeric proximity, limited our ability to resolve QTL in *Hort11-3*. Many of the horticultural QTL in this group appear to be coincident with *LFRes11-3* and *11-4* and *STRes11-4*, *11-5*, and/or *11-6*. However, this group also spans a large physical distance, approximately 45.8 Mbp of the total 56.3 Mbp attributed to chromosome 11, estimated from the cultivated tomato reference genome (SL2.5; Sol Genomics Network, http://solgenomics.net). If sufficiently large numbers (thousands) of recombinant progeny are screened, then there is opportunity via recombination to recover individuals with favorable linkages between resistance alleles and horticultural trait alleles.

Suppression of chromosomal recombination at and around centromeres has been observed in tomato (Pillen *et al.* 1996; Tanksley *et al.* 1992) and in other plant species such as potato (Tanksley *et al.* 1992), rice (Fan *et al.* 2011), and soybean (Du *et al.* 2012). It has been reported in other eukaryotes including *Drosophila melanogaster* (Beadle 1932), *Saccharomyces cerevisiae* (Lambie and Roeder 1986), and *Homo sapiens* (Mahtani and Willard 1998). Recombination suppression inhibits accurate QTL mapping, positional gene cloning, MAS, and identification of recombinant genotypes with favorable combinations of alleles that are originally linked in repulsion. For example, *fs8.1*, a major tomato fruit shape QTL differentiating fresh market and processing tomato market classes, was fine-mapped to a 3-Mbp centromeric region (Clevenger 2012; Ku *et al.* 2000) but has yet to be cloned. In contrast, even though Van Daelen *et al.* (1993) found genetic mapping of the centromeric *Mi* region of tomato chromosome 6 to be “rather cumbersome,” this nematode resistance gene was eventually cloned (Milligan *et al.* 1998).

QTL mapping and cloning in pericentromeric regions are further complicated by the presence of repetitive sequences. Pericentromeric heterochromatin in tomato contains a high frequency of repetitive DNA, primarily due to the presence of transposable elements (Wang *et al.* 2006). Repeated sequences prevent the placement of the unique oligonucleotide probes necessary for successful chromosome walking (Budiman *et al.* 2004). The reference genome sequence of cultivated tomato (SL2.5; Sol Genomics Network, http://solgenomics.net) may be helpful in overcoming this problem, because probes may be designed targeting nonrepetitive coding sequences interspersed within the pericentromeric heterochromatin. However, there is no publicly available genome sequence for *S. habrochaites*, and it is possible that the gene or genes underlying the QTL for resistance to *P. infestans* and horticultural traits within this region may be unique to *S. habrochaites*.

### Implications for tomato breeding

The complex genetic architecture of *P. infestans* resistance and horticultural traits within the region of chromosome 11 from *S. habrochaites* presents both opportunities and challenges for breeding applications of the resistance QTL and useful horticultural trait QTL. Challenges include linkage and/or pleiotropy between *P. infestans* resistance QTL and horticultural trait QTL, the pericentromeric location of these QTL, and the presence of QTL × E for some traits.

If unfavorable repulsion phase linkages of QTL are broken through recombination and recombinants are recovered, then the beneficial QTL alleles we have identified may prove useful for breeding tomato cultivars. In our study, we were able to separate some QTL alleles with negative effects from other desirable QTL alleles through recombination. However, because recombination is suppressed to less than 3% of the genome-wide average (750 kb per cM) (Tanksley *et al.* 1992) within the marker interval TG147–At5g04590 (Figure 2), it is likely that large numbers of progeny (thousands) will need to be screened to identify individuals with recombination between QTL within this region. In our present study, all loci with QTL × E have undesirable phenotypic effects when the *S. habrochaites* allele is present (Figure 1 and Table 4). If suitable environments and/or complementary genetic backgrounds for QTL deployment are identified, then negative effects may not preclude the use of the *P. infestans* resistance QTL linked to or pleiotropic to these horticultural QTL (Fleury *et al.* 2010; Lu *et al.* 2011).

The two sub-NILs with the highest *P. infestans* resistance (08GH3999 and 08GH8032) contained nonoverlapping *S. habrochaites* introgressions, consistent with our prior findings of multiple QTL for resistance within the *lb11b* region (Johnson *et al.* 2012). The level of resistance in these sub-NILs was comparable but their horticultural phenotypes differed, with 08GH8032 being significantly later-maturing than E6203, whereas 08GH3999 was not. Sub-NIL 08GH8032 also had shorter and wider FS, whereas 08GH3999 had taller and narrower FS. Both lines suffered a significant reduction in 30Wt, but yield was not significantly reduced in either line compared with E6203.

Sub-NIL 08GH3999 had the best combination of *P. infestans* resistance and horticultural trait phenotypes, and likely can be used directly as a donor line for MAS breeding to improve *P. infestans* resistance in tomato cultivars (Collard *et al.* 2005; Foolad and Panthee 2012). 08GH3999 can also be used as a parent for pyramiding resistance QTL alleles in crosses with other QTL donor lines (Collard and Mackill 2008; St. Clair 2010). Pyramiding of genes and QTL has been successful in many breeding efforts, including increasing barley stripe rust resistance (Castro *et al.* 2003), increasing barley yellow dwarf virus resistance (Riedel *et al.* 2011), and improving rice yield and quality (Wang *et al.* 2012). Sub-NIL 08GH3999 could be crossed with selected sub-NILs containing late blight disease resistance QTL from chromosome 5 to combine resistance QTL from two chromosomes (Haggard *et al.* 2013; Johnson *et al.* 2012) and potentially increase the level of quantitative resistance to *P. infestans*.

Additional research would be required to isolate the *P. infestans* resistance QTL in 08GH8032 from the linked horticultural QTL through recombination before it will be useful for tomato cultivar improvement. Based on the marker genotype of 08GH8032, its wild species introgression spans the centromere (S. Stack and L. Mueller, personal communication), so this work may be difficult, possibly requiring testing of thousands of progeny to recover favorable recombinants. Even so, the estimated large physical size of the *S. habrochaites* introgression in 08GH8032 suggests that obtaining desirable recombinants will be possible. Collectively, our results suggest that the resistance QTL alleles from *S. habrochaites* will eventually be useful for improving quantitative resistance to *P. infestans* in cultivated tomato.

## Supplementary Material

Supporting Information
