# Supplementary material for: Multiple QTL for Horticultural Traits and Quantitative Resistance to Phytophthora infestans Linked on Solanum habrochaites Chromosome 11
Source: G3 (Bethesda). 2014 Dec 12;5(2):219–33. doi: 10.1534/g3.114.014654 (PMC4321030; doi:10.1534/g3.114.014654)
Supplement: Supporting Information [file supp_5_2_219__index.html]

Supporting Information 

# Multiple QTL for Horticultural Traits and Quantitative Resistance to *Phytophthora infestans* Linked on *Solanum habrochaites* Chromosome 11

## Supporting Information for Haggard, Johnson, and St. Clair, 2015

**Files in this Data Supplement:**

- Table S1 - Genotypes and mean separations. (.xlsx, 83 KB)
- Table S2 - Horticultural trait Pearson correlation coefficients. (.xlsx, 24 KB)
- Table S3 - Raw phenotypic trait data. (.xlsx, 202 KB)
